# Stopping an outbreak of Pseudomonas aeruginosa ST111 by temporarily blocking the siphon to disinfect it before the transition to the wastewater drain

**DOI:** 10.3205/dgkh000630

**Published:** 2026-02-24

**Authors:** Torsten Stein, Martin Exner, Lisa-Marie Höfken, Katrin Schuldt, Axel Kramer

**Affiliations:** 1 BDH Greifswald, Center for Neurorehabilitation, Respiratory and Intensive Care Medicine and Clinic gGmbH Paraplegia, Germany; 2 Professor emeritus, Institute of Hygiene and Public Health, University of Bonn, Germany; 3 National Reference Centre for multidrug-resistant gram-negative bacteria for Germany; 4 Professor emeritus, Institute of Hygiene and Environmental Medicine, University Medicine Greifswald, Germany

**Keywords:** outbreak, carbapenem resistant P. aeruginosa, siphon disinfection, blocking the siphon

## Abstract

**Introduction:**

Resulting from an analysis of an outbreak of carbapenem-resistant *Pseudomonas (P.) aeruginosa*, the siphons of washbasins in patient rooms were identified as source of colonizations and/or infections. To avoid installing new siphons, a solution was needed to temporarily block the siphon before the transition to the wastewater drain.

**Result:**

By temporarily blocking the drain before the transition to the wastewater drain with a balloon, it was possible to disinfect the traps without disassembly and stop the outbreak.

**Discussion:**

Temporarily blocking the drain can stop outbreaks originating from the siphon. However, this does not protect the siphon from renewed contamination by patients, or subsequent biofilm formation. Therefore, the only option in cases of known patient colonization or infection with critical multi-resistant pathogens that can colonize the siphon is to disinfect the siphon after the patient’s discharge by temporarily blocking it. Since even non-resistant wastewater bacteria can cause infections, especially in immunocompromised patients, an option for obviating this problem would be to dispense with washbasins in high-risk areas such as units for intensive care, burns, cystic fibrosis and neonatology. As a general rule, water used to wash patients in bed must be disposed of in separate drains outside the patient's room. Since self-disinfecting siphons significantly reduce emissions from the siphon, combined with lower colonization or infection of exposed patients; their installation in high-risk areas may be useful for reducing nosocomial infections. However, in the event of an outbreak of a multi-resistant pathogen with the ability to colonize the siphon, it is necessary take samples of siphons and, if detected, to disinfect them by temporarily blocking them.

**Conclusion:**

The option of disinfection of siphons by temporarily blocking them is a technically simple, economically immediate measure.

## Zusammenfassung

**Einleitung:**

Im Ergebnis der Analyse eines Ausbruchgeschehens durch einen Carbapenem resistenten *P. aeruginosa* wurden die Siphons der Waschbecken in Patientenzimmern als Ursache für Kolonisationen bzw. Infektionen ermittelt. Um die Installation neuer Siphons zu vermeiden, sollte eine installatorische Lösung zur zeitweiligen Absperrung des Siphons vor dem Übergang zum Abwasserabfluss gefunden werden.

**Ergebnis:**

Durch zeitweilige Absperrung des Ablaufs vor dem Übergang zum Abwasserabfluss mit einem Ballon gelang es, die Siphons ohne Demontage zu desinfizieren und den Ausbruch zu beenden.

**Diskussion:**

Durch zeitweilige Absperrung des Ablaufs können Ausbrüche, ausgehend vom Siphon, beendet werden. Allerdings ist damit der Siphon nicht vor erneuter Kontamination durch den Patienten mit nachfolgender Biofilmbildung geschützt. Deshalb bleibt nur die Möglichkeit, bei bekannter Kolonisation oder Infektion des Patienten mit kritischen multiresistenten Erregern, die den Siphon kolonisieren können, den Siphon nach Entlassung des Patienten durch temporäre Absperrung zu desinfizieren. Da auch nicht resistente Erreger insbesondere bei abwehrgeschwächten Patienten Infektionen auslösen können, besteht eine Möglichkeit darin, auf Risikostationen, z.B. für Intensivtherapie, Verbrennungen, zystische Fibrose und Neonaten, auf Waschbecken zu verzichten. Grundsätzlich muss Wasser nach Patientenwaschung im Bett außerhalb des Patientenzimmers in separaten Ausgüssen entleert werden. Da durch selbstdesinfizierende Siphons die Emission aus dem Siphon deutlich reduziert wird, verbunden mit geringerer Kolonisation oder Infektion exponierter Patienten, kann deren Installation in Risikobereichen zur Reduktion nosokomialer Infektionen sinnvoll sein. In jedem Fall ist bei einem Ausbruch mit multiresistenten Erregern mit der Fähigkeit, den Siphonbereich zu kolonisieren, die Beprobung von Siphons vorzunehmen und im Fall des Nachweises die Desinfektion durch temporäre Absperrung durchzuführen.

**Schlussfolgerung:**

Die Möglichkeit der Siphondesinfektion durch zeitweilige Absperrung ist eine technisch einfach realisierbare, ökonomische Sofortmaßnahme, bis eine Umrüstung auf Waschbecken mit rückwärtigem Abfluss oder der Einbau von Siphons mit thermischer Desinfektion vorgenommen wird.

## Introduction

In 1991, Döring et al. [1] demonstrated that *Pseudomonas (P.) aeruginosa* can be transmitted from the siphon to the hands when the tap is opened due to aerosolization of the impinging water on the water standing in the siphon. More recently, siphons of both washbasins and shower drains have been repeatedly identified as a source of nosocomial transmission or outbreaks [2], [3], [4], [5].

Recurrent colonizations and infections caused by a multidrug-resistant (MDR) *Pseudomonas (P.) aeruginosa* strain of a carbapenemase (metallo-betalactamase) of type VIM-2 were observed over a period of 4 weeks in an intensive care unit for acute and early neurological rehabilitation. The first detection was in one patient on 20.01.2025. From April 6 to May 7 2025, the MDR *P. aeruginosa* strain was isolated from 9 patients. To determine the source, extensive environmental investigations were repeatedly carried out, considering the following sampling points: patient environment, beds, medical devices (perfusors, infusomats, oxygen sets), washbasins, bedpan sinks, removal area for gloves from glove boxes, disinfectant dispensers, disinfectant wipes, documentation systems, dispenser outlets, flexible nasoendoscopes, gastroscopes, drain sets and traps. As a result, the MDR *P. aeruginosa* strain was regularly detected in the siphons, while it was not detected at the other sampling points. Genotyping at the National Reference Center (NRZ) for Gram-negative hospital pathogens in Bochum, Germany, reconfirmed the identity of the *P. aeruginosa* isolates detected in the patients with the isolates from the siphons. The latter were already the initial focus as source of the outbreak, because colonizations and infections were limited to a few patient rooms. 

To assess the previous situation, it should be noted that a peracetic acid-based disinfectant (concentration >1,000 ppm in the solution used) was always added to the siphon as a preventive measure after each discharge of patients with infection or colonization with carbapenem-resistant Gram-negative bacteria.

Once the cause of the outbreak had been clarified, remediation was attempted as follows in accordance with the recommendation of the Commission for Hospital Hygiene and Infection Prevention (KRINKO) at the Robert Koch Institute on hygiene requirements for wastewater-carrying systems in medical facilities [6]. The traps were replaced with new traps. After thorough mechanical cleaning, the washbasins, including the closures, were disinfected with a peracetic-acid based disinfectant. Similarly, the accessible pipe parts downstream the trap were cleaned and disinfected. Despite these measures, the new traps were recolonized with the outbreak strain within 3 days. Therefore, a solution had to be found to fill the siphon with disinfectant solution for the duration of the exposure time for the disinfectant until the transition to the wastewater pipe.

## Method

**Figure 1:**
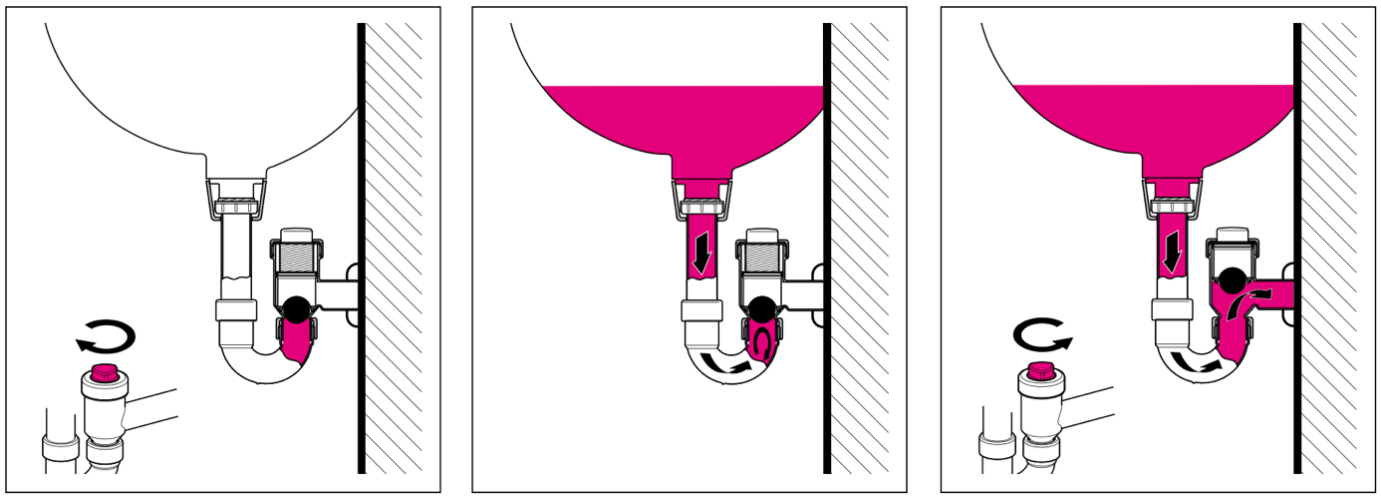
Washbasin drain. a) Close the valve by turning it to the right; b) fill with disinfectant solution; c) open the valve by turning it to the left.

The temporary blocking of the siphon before the transition to the wastewater drain was achieved with an installed balloon, which blocks the siphon to the wastewater drain for the duration of the disinfection. This option is available as a commercially available product (Geberit, International AG, Jona, Switzerland). In daily use, the installed siphon remains open to allow the water to drain off. However, to fill with the disinfectant solution, the screw-on cap at the back which allows draining is turned to the right to close it (Figure 1a (Fig. 1)). 

Once the disinfectant solution has been poured in (Figure 1b (Fig. 1)) and let sit for the required exposure time, the screw-on cap is turned back to the left (opened; Figure 1c (Fig. 1)) so that water drainage can resume.

## Results

The temporary blocking of the siphon and disinfection succeeded in immediately stopping the outbreak. No new colonizations or infections occurred after disinfection of the blocked siphon. The outbreak strain was no longer detectable in the siphon water.

## Discussion

The siphon is an open reservoir of pathogens from patients’ flora. When water enters, bacteria are ejected from the wastewater standing in the siphon over a radius of up to 1.50 m (authors’ measurement). In cases of siphon contamination of >10^5^ colony-forming units (cfu)/mL, the transmission of bacteria to the hands of nursing staff during hand washing has been demonstrated [1]. In siphons, the genera *Pseudomonas, Acidovoras, Diaphorobacter, Acinetobacter, Rhizobium, Citrobacter, Aeromonas*, and *Klebsiella* were represented in a stable microbiome and resistome, that is, the drainome [7]. MDR *P. aeruginosa*, e.g., VIM-producing species, were repeatedly detected [8]. Pulmonary non-tuberculous mycobacteria (NTB) have been detected in household plumbing as possible cause of chronic rhinosinusitis [9]. Siphons have been the source of both individual infections [10] and nosocomial outbreaks. Frequently identified outbreak strains were carbapenemase-producing Enterobacteriaceae (e.g. *Klebsiella pneumoniae, Escherichia coli, Enterobacter* spp.), nonfermenting Gram-negatives (e.g., *Pseudomonas, Burckholderia, Acinetobacter, Stenotrophomonas* and *Serratia* spp.) [10], [11], [12], [13], [14], [15], [16], [17], [18]. 

The ST111 sequence type isolated here was detected during an outbreak in sink drains, two toilets, and a cleaning bucket. The outbreak containment was achieved by replacing U-bends, and cleaning buckets, and switching from quaternary ammonium compounds to oxygen-releasing disinfectant products [19]. This sequence type was the cause of another outbreak in Germany, with confirmed sink-to-patient transmission related to sink drains in two ICU rooms [20].

Due to the risk of nosocomial infections caused by siphon contamination, water-free patient care is increasingly being chosen as an alternative, at least in high-risk areas such as intensive care units and neonatal ICUs, resulting in a reduction in colonization and infections caused by Gram-negative bacteria [21], [22].

To stop an outbreak originating in the siphon, the following immediate measures were effective: cleaning the siphon 3 times a day and changing the siphon, after which an outbreak that had existed for 5 years was ended [18]. 

In contrast, siphon disinfection, e.g., with peracetic acid, only has a short-term effect, because the biofilm in the upper section of the drain system remained unaffected, and within two days the liquid in the P-traps was recolonized at a concentration >10^5^ cfu/mL. After one month, this disinfecting protocol had not achieved a lasting decontamination of the sink drains [8]. A foam product containing 3.13% hydrogen peroxide and 0.05% peracetic acid resulted in significantly reduced recovery of Gram-negative bacilli on days 1, 2, and 3 after treatment, with a return to baseline by day 7 [23]. Similarly, in a longitudinal study with analysis of the drainome, elimination of critical MRE Gram-negatives was not attainable, leading to the conclusion that the optimal strategy for reduction of transmission from sinks is not known [7]. 

Installation of self-disinfecting sink systems (SDSS) with thermal disinfection and a biofilm-inhibiting inner surface was found to prevent pathogen reflux from the washbasin siphon trap into the washbasin. The marker organism used was a MDR *P. aeruginosa*. During a six-month observation period following the introduction of the self-disinfecting sink drains, no instance of nosocomial colonization of patients with a carbapenemase resistant *P. aeruginosa* was noted [24]. SDSS were superior to sink replacements in preventing emissions from aerosol pathogens but were unable to completely prevent the emission [25]. After replacing sink drains by SDSS the rates of microbial colonization of patients as well as the rates of incidents due to nosocomial infections were decreased [26]. Therefore, SDSS can be advantageous in special units, e.g., for cystic fibrosis patients or intensive-care neonatology. 

## Conclusion

The recolonization of the siphon after routine flushing of the siphon with disinfectant after each discharge of a patient who was colonized by or infected with the outbreak strain indicates that eradication of the outbreak strain from the siphon is not possible, as the strain was always detectable in subsequent sampling. 

To stop the outbreak, the siphon disinfection by temporarily blocking the siphon to the wastewater is a technically simple and effective immediate measure and should be performed after each discharge of a carrier of a multidrug resistant Gram-negative bacterial species, unless the wash water is disposed of in a separate drain.

## Notes

### Author’s ORCID

- Kramer A: https://orcid.org/0000-0003-4193-2149

### Funding

None. 

### Acknowledgments

The authors would like to thank Geberit International AG, Jona, Switzerland, for permission to use the figure and Mr. Karsten Nietz for installing the siphon blocking system. 

### Competing interests

The authors declare that they have no competing interests.
